# Do We Notice when Communication Goes Awry? An Investigation of People's Sensitivity to Coherence in Spontaneous Conversation

**DOI:** 10.1371/journal.pone.0103182

**Published:** 2014-07-29

**Authors:** Bruno Galantucci, Gareth Roberts

**Affiliations:** 1 Psychology Department, Yeshiva University, New York, New York, United States of America; 2 Haskins Laboratories, New Haven, Connecticut, United States of America; The University of Nottingham, United Kingdom

## Abstract

In the dominant theoretical framework, human communication is modeled as the faithful transmission of information. This implies that when people are involved in communicational exchanges, they should be sensitive to the success with which information is transmitted, easily detecting when conversations lack coherence. The expectation that humans are good at detecting conversational incoherence is in line with common intuition, but there are several reasons to suspect that it might be unrealistic. First, similar intuitions have been shown to be unrealistic for a number of psychological processes. Second, faithful information transmission may conflict with other conversational goals. Third, mechanisms supporting information transmission may themselves lead to cases of incoherence being missed. To ascertain the extent to which people are insensitive to patches of serious conversational incoherence, we generated such patches in the laboratory by repeatedly crossing two unrelated conversations. Across two studies, involving both narrowly and broadly focused conversations, between 27% and 42% of the conversants did not notice that their conversations had been crossed. The results of these studies suggest that it may indeed be unrealistic to model spontaneous conversation as faithful information transmission. Rather, our results are more consistent with models of communication that view it as involving noisy and error-prone inferential processes, serving multiple independent goals.

## Introduction

*The single biggest problem in communication is the illusion that it has taken place.*

(attributed to George Bernard Shaw)

For more than half a century, the study of human communication has been dominated by what has been referred to as the *code model* [1], according to which communicative exchanges are to be understood in terms of the faithful transmission of information from a source to a receiver [2]. This assumption implies that, when humans communicate, they should be sensitive to evidence of transmission problems and respond accordingly. On the basis of the code model, then, one should expect cases of blatant incoherence in conversation to be readily noticed.

This expectation of sensitivity to incoherence is very much in line with common intuition. To assess the degree to which that is the case, we presented a scenario to a random sample of 20 college students and professors in which an individual, while engaged in a conversation with Partner A, suddenly starts talking as if engaged in an unrelated conversation with Partner B. All 20 respondents thought that the resulting incoherence would be disruptive and noticed by Partner A. However, as the epigraph suggests, this intuition about communicative precision may be illusory; indeed, there are several reasons to suppose that even such dramatic cases of incoherence might be missed or ignored. First, similar intuitions about the importance of faithful information transmission in human psychological processes have been shown to be unrealistic in a number of apparently straightforward perceptual tasks [3]–[6]. Second, it has long been recognized that human communication is not devoted solely to the faithful transmission of information. In particular, it is also used for what have been termed *phatic* purposes [7], that is, for the purpose of creating and maintaining social relationships (cf. [8]). This may conflict with faithful information transmission. For example, conversational repairs undertaken for the sake of informational coherence can have negative social consequences and are thus often avoided [9]. Finally, it may be the case that the faithful transmission of information is undermined by the very mechanisms that support effective communication. According to Gricean models of communication, a speaker's meaning is reconstructed through an inferential process involving expectations of relevance [10], [11]. Such expectations may lead listeners to explain away incoherencies, obscuring genuine cases of miscommunication.

To ascertain the extent to which conversants are indeed insensitive to informational incoherence in spontaneous conversation, we measured whether people would detect patches of blatant incoherence while chatting. Specifically, we asked pairs of participants to chat about a cartoon using instant-messaging software. We generated incoherence in these conversations by repeatedly crossing them with different conversations in which other pairs were chatting about a different cartoon, creating scenarios like the one presented to the sample described above. At the end of the chat we asked participants if they had noticed the crossings.

## STUDY 1: Incoherence detection in narrowly focused conversation

### Methods

#### Participants

20 native-English-speaking students, with no deficits in color vision or communicative ability, participated for $20 each.

#### Ethical statement

Ethical approval was granted by the Institutional Review Board at Yeshiva University. All participants gave written consent to participate.

#### Procedure

**Figure 1:**
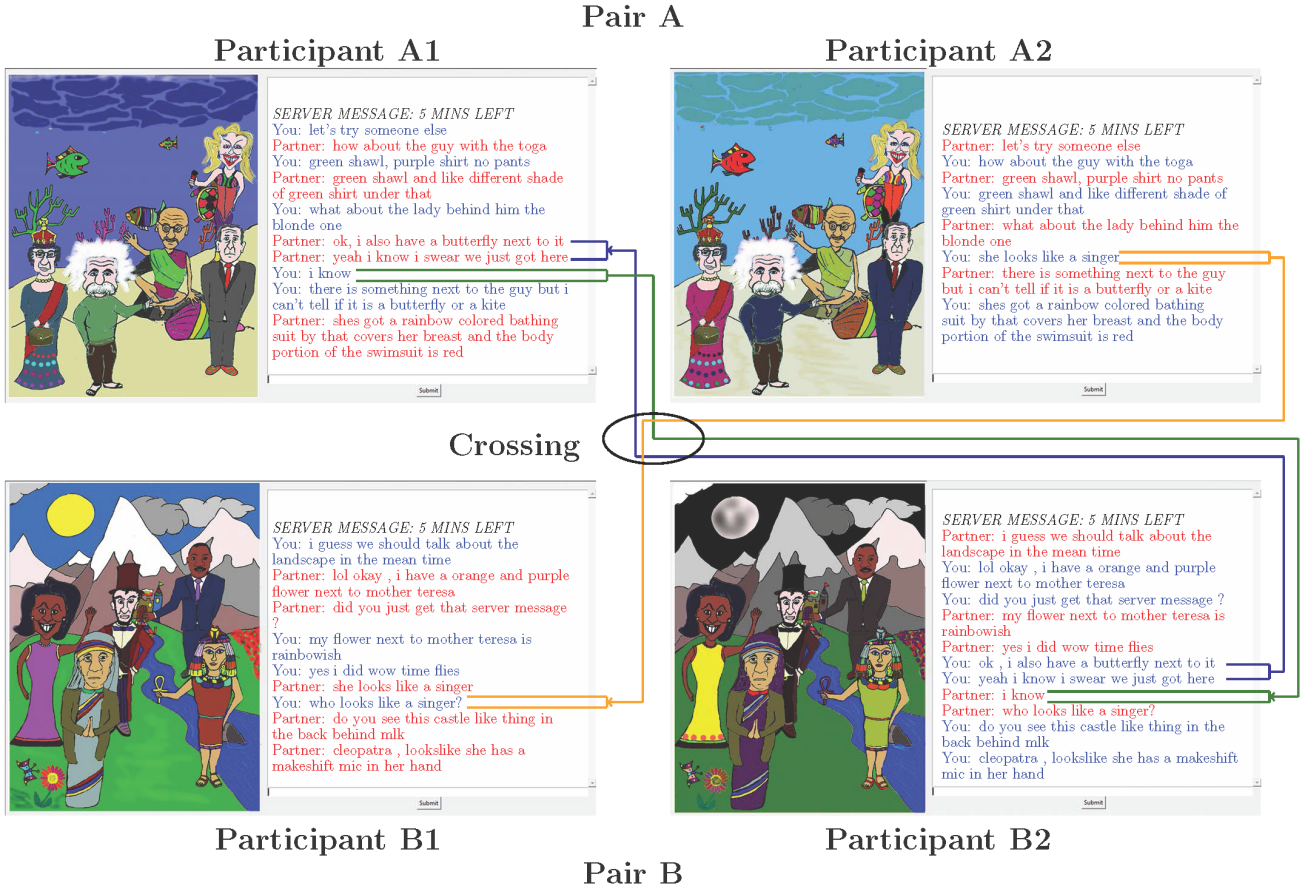
Diagram of the concurrent conversations with crossed utterances highlighted.

Participants took part in pairs. Care was taken that the members of a pair did not already know each other and that they met only at the start of the study. On arriving at the lab, members of a pair were seated in separate rooms and received the same scripted instructions (see Materials S1). Each member of a pair sat in front of a computer displaying a cartoon of five famous people and an instant-messaging window (Figure 1). As in widely used instant-messaging programs, the messaging window consisted of two parts. At the bottom was a space where a participant could type messages and relay them to their partner. The rest of the window consisted of a space in which messages would appear. Messages the participant had sent appeared in black and were preceded by “You:”; received messages appeared in red and were preceded by “Partner:”. Both members of the pair saw the same exact cartoon except that it was colored differently for each of them. (This was explained to both participants.) They were then given the task of identifying the differences by using the instant-messaging software to chat for 15 minutes.

Although the above account focuses on one pair only, each trial in fact involved two pairs, Pair A and Pair B. The two pairs performed the same task simultaneously, but with different cartoons. Pair A's cartoon contained five different famous people from Pair B's cartoon, set against a very different background (Figure 1). The software ensured that the task began at the same time for both pairs, but no participant was aware of the existence of the other pair. Over the course of each conversation there were four 30-second *crossings* during which each member of Pair A was re-partnered with a random member of Pair B. Participants were not warned before the study that these crossings would occur (indeed, no participant was even aware of the existence of the other pair), and there were no markers to indicate that they were occurring. All messages received, whoever had sent them, continued to appear in red preceded by “Partner:” (Figure 1). Crossings occurred at random points but began at least three minutes into the conversation.

After 15 minutes had elapsed the chat window closed and a new window presented the following questions one by one:

1. How did you find the conversation today?
2. Did the conversation go smoothly?
3. Did you ever feel like you were having trouble communicating with your partner?
4. Did you notice anything unusual in the conversation?
5. Participants in this study are put in one of two groups. 50% of participants are put in the No-Crossing Group. If we put you in the No-Crossing Group then all the messages you received came from your partner. The other 50% of participants are put in the Crossing Group. If we put you in the Crossing Group then some of the messages you received came from a different participant who intended them for someone else and did not know that they would come to you. Which group do you think you were in? Note: If you are correct, you will win $3!

Participants who answered “no” to Question 2 or “yes” to Question 3 or 4 were asked to explain their answers before the next question appeared.

### Results

Although our investigation focused on Question 5, we used Questions 2–4 to enhance the precision of our analysis, excluding participants whose answers to Question 5 was unlikely to originate from a genuine assessment of the conversation. We adopted a very conservative criterion, excluding only participants whose answers to Question 2–4 were all inconsistent with their answer to Question 5. There were two possible ways participants could fulfill this criterion. On the one hand, participants could guess that they belonged to the Crossing Group and yet report (a) that their conversation had gone smoothly, (b) that they had had no trouble communicating with their partner, and (c) that they had not noticed anything unusual. We will refer to such participants as *Inconsistent Detectors*. On the other hand, participants could guess they did not belong to the Crossing Group and yet report (a) that their conversation had not gone smoothly, (b) that they had had trouble communicating with their partner, and (c) that they had noticed something unusual. We will refer to such participants as *Inconsistent Non-detectors*.

One participant was an Inconsistent Detector and there were no Inconsistent Non-detectors. This suggests the existence of a bias toward suspecting unusual circumstances (a common bias in psychology experiments; cf. [12], [13]). To test if such a bias was indeed present, we replicated Study 1 with 10 new participants and no crossings (i.e., each participant chatted with the same partner for the entire session). Consistent with the presence of a bias toward suspecting unusual circumstances, four participants (40%) incorrectly answered that they were in the Crossing Group.

The one Inconsistent Detector was excluded from all analyses reported below.

#### Crossing detection rates

**Figure 2:**
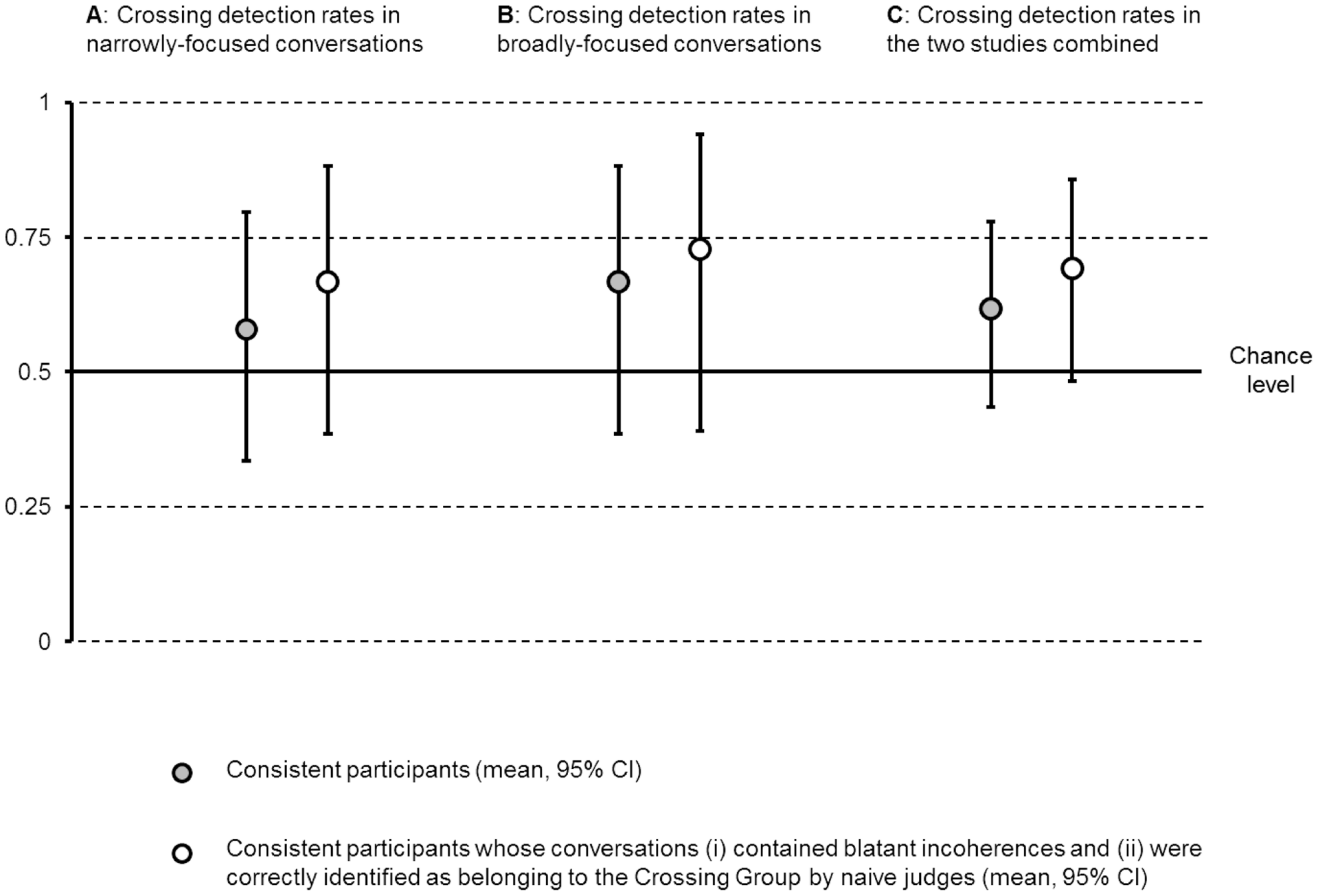
Crossing detection rates in (A) Study 1, (B) Study 2, and (C) Studies 1–2 combined.

11 out of 19 participants answered that they were in the Crossing Group, leading to a crossing detection rate of 57.9% (Figure 2a). This rate is not significantly different from chance: 95% CI [33.5%, 79.7%] (all CIs presented in this paper were computed using the Clopper–Pearson exact method). It is also rather far from the expectations of the sample reported in the introduction.

The finding that over 40% of participants failed to noticed that the conversation they were holding was repeatedly crossed with a different conversation seems to suggest that we are surpr“‘isingly insensitive to conversational incoherence. However, there could be an alternative explanation for our results. The fact that the conversations were repeatedly crossed with each other is not in itself a guarantee that the participants were exposed to clear evidence that these crossings had occurred. Furthermore, the task of finding color differences might have focused the conversations so narrowly that it reduced potential differences between them, masking the crossings. We dealt with these possibilities as follows. First, we conducted two different analyses of the conversational transcripts to determine whether participants had indeed been exposed to clear evidence of crossings. These analyses are presented in the next section. Second, we replicated the study with a much broader conversational focus. This replication is presented below as Study 2.

#### Crossing detection rates for blatantly crossed conversations

In order to ascertain whether participants were indeed exposed to blatantly crossed conversations, we analyzed the transcripts of the conversations in two ways.

First, we read the transcripts, looking for out-of-the blue references to famous people or salient items which were not present in the cartoon seen by the pair and had not been mentioned earlier in the conversation. Considering that both members of every pair saw the same cartoon as each other and knew that this was the case, such references provided blatant indications that the conversation had been crossed with a different one. We found that all but three participants were exposed to at least one such blatant indication.

The second analysis relied on naive judges. Specifically, four judges were given ten transcripts each, five from the No-Crossing Group (these were the five conversations from the control study reported above, in which there were no crossings) and five from the Crossing Group. In the No-Crossing Group every participant's transcript was identical to their partner's, meaning that there were only five distinct transcripts, which were given to all judges. In the Crossing Group, by contrast, no two transcripts were identical. This means that there were twenty distinct transcripts, which can be divided into five sets, each including four different transcripts from pairs whose conversations were crossed with each other. These transcripts were distributed across judges in such a way that (a) every judge received one transcript from each set, and (b) each transcript was given to one judge.

We explained the basics of the study to the judges and asked them to decide which of the ten transcripts they had been given came from pairs in the No-Crossing group and which came from pairs in the Crossing group. Judges were paid $10 an hour for their time, or given course credit (in either case the reward was doubled if discrimination was perfect). There was no time-limit for completing the task.

The four naive judges were very accurate in performing the task, misclassifying only one of the 20 transcripts from the Crossing group.

To restrict the analysis presented above to those cases where the crossings had clearly achieved their intended goal, we re-ran it excluding the three participants who were not exposed to blatant indications of crossings as well as the one participant whose conversation was incorrectly classified by a judge.

Ten of the remaining 15 participants answered that they were in the Crossing Group, leading to a crossing detection rate of 67% (Figure 2a). This rate is not significantly different from chance (95% CI [38.4%, 88.2%]) and is rather far from the expectations of the sample reported in the introduction. In other words, the more stringent analysis presented here is consistent with the analysis presented above: People are surprisingly insensitive to conversational incoherence.

## STUDY 2: Incoherence detection in broadly focused conversation

As noted above, the narrow conversational focus in Study 1 might have reduced the differences between conversations, masking the crossings. Such an explanation seems unlikely for two reasons. First, pairs discussed cartoons with non-overlapping groups of famous people set against strikingly different backgrounds (Figure 1). In the majority of cases, as shown by the analysis of the transcripts, this led to blatant violations of conversational coherence. Second, the naive judges in Study 1 were almost perfect in identifying which conversations had been crossed. However, to investigate whether we would find the same results with more broadly focused conversations, we replicated Study 1 with a new task. This study was identical to Study 1 except that the participants' task was to discuss which of the five famous people they would most and least like to spend a day with (see Materials S2).

### Methods

#### Participants

20 native-English-speaking students, with no deficits in color vision or communicative ability, participated for $20 each. These participants were different from the 20 participants of Study 1 and did not know them or know of the existence of the previous experiment.

#### Ethical statement

Ethical approval was granted by the Institutional Review Board at Yeshiva University. All participants gave written consent to participate.

#### Procedure

The procedure was identical to that of Study 1, except that participants were given the task of discussing which of the five famous people they would most and least like to spend a day with.

### Results

Four participants were Inconsistent Detectors and one participant was an Inconsistent Non-detector. As in Study 1, this suggests the existence of a bias toward suspecting unusual circumstances. To test if such a bias was also present in Study 2, we replicated it with 10 new participants and no crossings (i.e., each participant chatted with the same partner for the entire session). Consistent with the presence of a bias toward suspecting unusual circumstances, two participants (20%) incorrectly answered that they were in the Crossing Group.

The five inconsistent participants were excluded from all analyses reported below.

#### Crossing detection rates

10 out of 15 participants answered that they were in the Crossing Group, leading to a crossing detection rate of 66.7% (Figure 2b). This rate is not significantly different from chance (95% CI [38.4%, 88.2%]) and is rather far from the expectations of the sample reported in the introduction.

#### Crossing detection rates for blatantly crossed conversations

In order to ascertain whether participants were indeed exposed to blatantly crossed conversations, we analyzed the transcript of the conversations in the same two ways as in Study 1. Based on the first analysis, all but three participants were exposed to at least one blatant indication that a crossing had occurred. For the second analysis, the four naive judges from Study 1 were recruited again to perform the same task as before with the transcripts from Study 2. Again they were rather accurate, misclassifying only four of the 20 transcripts from the Crossing group.

To restrict the analysis presented above to those cases where the crossings had clearly achieved their intended goal, we re-ran it excluding the three participants whose conversations contained no blatant indication of the crossings as well as three participants whose conversations were misclassified by the judges (the fourth participant had already been excluded as an Inconsistent Non-detector). This led to the exclusion of four participants in total, as two participants fulfilled both criteria.

Eight of the remaining 11 participants answered that they were in the Crossing Group, leading to a crossing detection rate of 72.7% (Figure 2b). This rate is not significantly different from chance (95% CI [39%, 94%]) and is rather far from the expectations of the sample reported in the introduction.

Although the detection rates in Study 2 were higher than in Study 1, its results are consistent with the core results of Study 1: People are surprisingly insensitive to conversational incoherence, even when their conversation is freed from tight task constraints.

## Discussion

Across the two studies, between 27.3% and 42.1% of our participants (depending on the analysis) failed to notice that a conversation they were having was repeatedly crossed with an unrelated one. Indeed, detection rates were never significantly better than chance in the two studies, and this surprising finding is confirmed by an analysis of their combined results: 21 out of 34 consistent participants in the two studies answered that they were in the Crossing Group, leading to a crossing detection rate of 61.8% (95% CI [43.6%, 77.8%]; Figure 2c). Excluding participants whose conversations contained no blatant indication of the crossings or were misclassified by the naive judges did not change the finding: 18 out of the 26 remaining participants answered that they were in the Crossing Group, leading to a crossing detection rate of 69.2% (95% CI [48.2%, 85.6%]; Figure 2c). Altogether this is striking evidence that, although people can and do notice incoherence in conversation, they often fail to do so, even when such incoherence is blatant.

This result is at odds with what one would expect on the basis of the code model [1]. It should not, however, be taken to mean that the code model never applies. When landing a plane, for instance, the faithful transmission of information between control tower and pilot is of the utmost importance. However, the very fact that such interactions are tightly constrained by strict protocols suggests that, far from being a natural mode of human communication, the faithful transmission of information is a highly effortful undertaking. In other words, the results of our study are more consistent with models of communication that view it as noisy and error-prone [14], [15], as relying on assumptions of relevance and inferential processes with multiple inputs [10], [11], and as serving goals other than the faithful transmission of propositional information [7], [8]. On this basis, we should consider the possibility that failures of communication are rather more common than we tend to assume (cf. [9]).

Our method may also provide new opportunities for research. Until now, researchers investigating failures in communication have relied on naturalistic observations [9], or experiments involving contrived conversations [16] or confederates [17]. Manipulating online conversation (cf. [18]) opens new doors for investigating human interaction.

## Supporting Information

Materials S1 **Instructions for participants (study 1).**
(PDF)

Materials S2 **Instructions for participants (study 2).**
(PDF)

## References

[1] Blackburn PL (1999) The Code Model of Communication: A Powerful Metaphor in Linguistic Metatheory. Ph.D. thesis. Retrieved from ProQuest Dissertations & Theses Full Text database (304574107).

[2] Shannon CE, Weaver W (1949) The mathematical theory of communication. Urbana, IL: University of Illinois Press.

[3] Rensink RA, O'Regan JK, Clark JJ (1997) To see or not to see: The need for attention to perceive changes in scenes. Psychological Science 8: 368–373.

[4] Simons DJ, Levin DT (1998) Failure to detect changes to people during a real-world interaction. Psychonomic Bulletin and Review 5: 644–649.

[5] Simons DJ, Chabris CF (1999) Gorillas in our midst: Sustained inattentional blindness for dynamic events. Perception 28: 1059–1074. 10694957 10.1068/p281059

[6] Fenn KM, Shintel H, Atkins AS, Skipper JI, Bond VC, et al (2011) When less is heard than meets the ear: Change deafness in a telephone conversation. The Quarterly Journal of Experimental Psychology 64: 1442–1456. 21604232 10.1080/17470218.2011.570353

[7] Malinowski B (1923) The problem of meaning in primitive languages. In: Ogden CK, Richards IA, editors, The Meaning of Meaning. London: Routledge. pp. 146–152.

[8] Dunbar RIM (1996) Grooming, Gossip and the Evolution of Language. London: Faber and Faber.

[9] Tzanne A (2000) Talking at Cross-Purposes: The Dynamics of Miscommunication. John Benjamins.

[10] Grice HP (1975) Logic and conversation. In: Cole P, Morgan JL, editors, Syntax and Semantics, Vol. 3: Speech Acts. New York: Academic Press. pp. 41–58.

[11] Wilson D, Sperber D (2004) Relevance theory. In: Horn LR, Ward G, editors. Handbook of Pragmatics. Oxford: Blackwell. pp. 607–632.

[12] Kelman HC (2007) Human use of human subjects: The problem of deception in social psychological experiments. Psychological Bulletin 67: 1–11. 10.1037/h0024072 6035775

[13] MacCoun RJ, Kerr NL (1987) Suspicion in the psychological laboratory: Kelman's prophecy revisited. American Psychologist 42: 199.

[14] Reddy MJ (1979) The conduit metaphor: A case of frame conict in our language about language. Metaphor and thought 2: 164–201.

[15] Keysar B (2007) Communication and miscommunication: The role of egocentric processes. Intercultural Pragmatics 4: 71–84.

[16] Keysar B, Henly AS (2002) Speakers' overestimation of their effectiveness. Psychological Science 13: 207–212. 12009039 10.1111/1467-9280.00439

[17] Keysar B, Lin SH, Barr DJ (2003) Limits on theory of mind use in adults. Cognition 89: 25–41. 12893123 10.1016/s0010-0277(03)00064-7

[18] Healey PGT, Purver M, King J, Ginzburg J, Mills G (2003) Experimenting with clarification in dialogue. In: Proceedings of the 25th Annual Conference of the Cognitive Science Society. Boston: Cognitive Science Society, pp. 539–544.

