# Supplementary material for: Do We Notice when Communication Goes Awry? An Investigation of People's Sensitivity to Coherence in Spontaneous Conversation
Source: PLoS One. 2014 Jul 29;9(7):e103182. doi: 10.1371/journal.pone.0103182 (PMC4114551; doi:10.1371/journal.pone.0103182)
Supplement: Materials S1 — Instructions for participants (study 1). (PDF) [file pone.0103182.s001.pdf]

Instructions for participants
(Study 1)

You're about to take part in an experiment in which you will chat to your partner using an instant-messenger program.

On the left of the screen you'll see a group of famous people. On the right of the screen you'll see the messenger window, where you can type messages to your partner.

Your partner will see the same group of famous people as you in every respect except that the colors will be different. Your task is to communicate with your partner and find all the color differences.

The chat program will start working, and the picture will appear, when both you and your partner have clicked Start. You will have fifteen minutes to chat, and then there will be a short questionnaire.

Do you have any questions?
