# Supplementary material for: Do We Notice when Communication Goes Awry? An Investigation of People's Sensitivity to Coherence in Spontaneous Conversation
Source: PLoS One. 2014 Jul 29;9(7):e103182. doi: 10.1371/journal.pone.0103182 (PMC4114551; doi:10.1371/journal.pone.0103182)
Supplement: Materials S2 — Instructions for participants (study 2). (PDF) [file pone.0103182.s002.pdf]

# Instructions for participants
# (Study 2)

You're about to take part in an experiment in which you will chat to your partner using an instant-messenger program.

In the left of the screen you'll see a group of famous people. On the right of the screen you'll see the messenger window, where you can type messages to your partner.

Your partner will see the same group of famous people. Your task is to chat with your partner about these famous people. In particular, if you could spend a day of your life with one of them, who would you choose? Who would you least like to spend a day with?

The chat program will start working, and the picture will appear, when both you and your partner have clicked Start. You will have fifteen minutes to chat, and then there will be a short questionnaire.

Do you have any questions?
